# Antimicrobial Polymers with Metal Nanoparticles

**DOI:** 10.3390/ijms16012099

**Published:** 2015-01-19

**Authors:** Humberto Palza

**Affiliations:** Departamento de Ingeniería Química y Biotecnología, Facultad de Ciencias Físicas y Matemáticas, Universidad de Chile, Beauchef 850, Santiago 8320000, Chile; E-Mail: hpalza@ing.uchile.cl; Tel.: +56-22-978-4085; Fax: +56-22-699-1084

**Keywords:** antimicrobial metals, polymer nanocomposites, copper, silver

## Abstract

Metals, such as copper and silver, can be extremely toxic to bacteria at exceptionally low concentrations. Because of this biocidal activity, metals have been widely used as antimicrobial agents in a multitude of applications related with agriculture, healthcare, and the industry in general. Unlike other antimicrobial agents, metals are stable under conditions currently found in the industry allowing their use as additives. Today these metal based additives are found as: particles, ions absorbed/exchanged in different carriers, salts, hybrid structures, *etc*. One recent route to further extend the antimicrobial applications of these metals is by their incorporation as nanoparticles into polymer matrices. These polymer/metal nanocomposites can be prepared by several routes such as *in situ* synthesis of the nanoparticle within a hydrogel or direct addition of the metal nanofiller into a thermoplastic matrix. The objective of the present review is to show examples of polymer/metal composites designed to have antimicrobial activities, with a special focus on copper and silver metal nanoparticles and their mechanisms.

## 1. Introduction

Non-essential metals, such as silver, can be toxic to bacteria, having biocidal activities at exceptionally low concentrations, while essential metals, such as copper, can also be lethal above some threshold despite their relevance in the biochemistry of organisms [1]. Because of this biocidal activity, metals have been widely used for centuries as antimicrobial agents in agriculture, healthcare, and industry in general. Metal, oxide, or salt compounds based on copper and silver are among the most widely applied antimicrobial agents in this context [2]. However, the use of these metals in industrial applications presents several challenges associated with the nature of the metal itself. Consequently, one of their first applications was in the form of salt-based additives, for instance as silver nitrate, avoiding its highly expensive metal form. Metal copper otherwise is cheaper than silver but presents corrosion processes at standard conditions. The processing and manipulation of metal-based materials, such as alloys, is another issue that should be resolved. Therefore, these metals are still mostly used as additives in several applications such as wood preservation, antifouling paints, and antibacterial textiles, among others. However, with the recent developments in materials science, their uses can be extended today to metal surfaces and coatings, chelates, and nanomaterials [1]. Metal nanoparticles are stressed due to both their enhanced antimicrobioal behaviour as compared with traditional materials and their capacity to be embedded into polymer matrices.

Polymer/metal composites emerge as a route to further extend the applications of biocide metals as a large percentage of hospital acquired infections (HAIs) are spread through surface contacts or catheters mostly made of plastics. For instance, around 80%–95% of hospital-acquired urinary tract infections originate from urinary catheters [3,4]. This is even more relevant taking into account the steady growth of the polymer market, especially that based on polyolefins (*i.e.*, polypropylene and polyethylene), in healthcare applications. This growth is due to its properties such as: chemical, radiation, and heat resistance, stiffness, clarity, barrier behavior for gases and liquids, impact, flexibility and moderate cost [5]. For instance, polypropylene (PP) products are extensively used in medical devices, packaging products, and delivery systems for solid and liquid pharmaceuticals [5]. About a quarter of disposable and a third of non-disposable medical devices are PP-based, while the global growth rate for PP in the healthcare industry is about 8.7% per year [6]. Polyamide is another example of the relevance of polymers in healthcare applications as wound sutures, artificial tendons, and medical packaging are made from this thermoplastic matrix [7].

Antimicrobial polymers therefore are highly demanded as a strategy to avoid HAIs and they can be prepared either by embedding a biocide agent into the polymer bulk, for instance, during their processing or by applying surface coatings [8,9,10,11]. For instance, either electrochemical or plasma based methods have been recently applied to produce antimicrobial polymer/metal composite coatings [9,12,13,14,15]. A different approach is the polymerization of monomer-containing biocide groups or the grafting of antimicrobial agents into the polymers [11]. The polymerization of the biocide polymer on the surface of commercial polymers by atom transfer radical polymerization has been also reported [16,17]. From all these methods, the direct addition of the biocide agent into the polymers has received considerable attention especially for thermoplastics such as polyolefins [18]. The main reason is that this method can be easily implemented in the standard processing units already designed to prepare particulate filled polymer composites and that are used extensively in the industry [19]. In this context, polymer/metal composites prepared by melt blending are perceived as a useful way to produce biocidal polymers [1]. Moreover, metals do not suffer degradation under the standard processing conditions of thermoplastic polymers (~200 °C) [18,20]. This approach can be further extended to antimicrobial metal nanoparticles as it seems that similar to other nanoparticles applications, their addition into polymer matrices could be the fastest route to take commercial advantage of their enhanced properties.

## 2. Antimicrobial Metals

### 2.1. General Aspects

**Figure 1:**
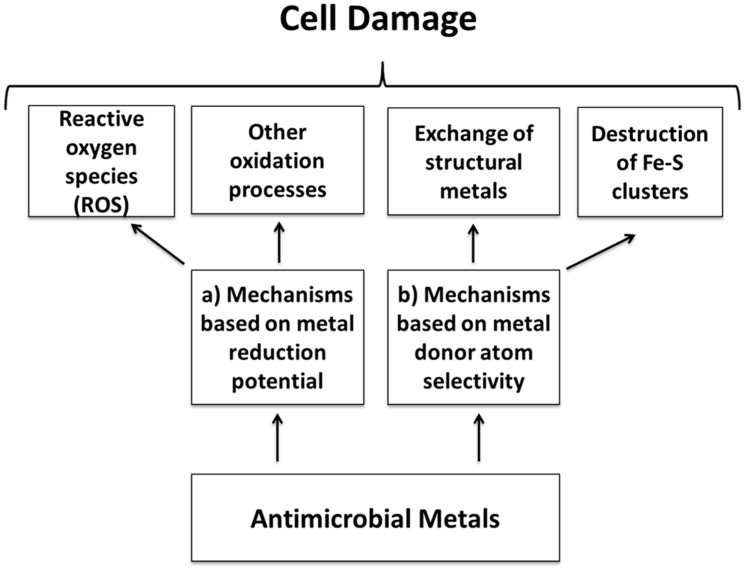
A summary of the main mechanisms behind the antimicrobial behavior of metal as separated according to the specific metal property responsible for this action: (**a**) reduction potential and (**b**) donor atom selectivity and/or speciation.

Metal can be extremely toxic to most bacteria and yeast at exceptionally low concentrations [1]. Because of this biocide activity, some particular metals have been used as antimicrobial agents since ancient times. For instance, vessels made of Cu and Ag have been used for water disinfection and food preservation since the time of the Persian kings [1]. This practice was later adopted by the Phoenicians, Greeks, Romans and Egyptians. Despite this long evidence, today the specific mechanisms explaining the toxicity of metals are not yet fully elucidated as several variables are involved. However, depending on the metal property, the biocide behavior can be triggered by: (a) the metal reduction potential and (b) the metal donor atom selectivity and/or speciation. Based on this, Figure 1 shows an overview of the different mechanisms behind metal toxicity as explained below in detail.

(a) Mechanisms based on the reduction potential of metals: An important characteristic of metals is their capacity to participate in redox reactions determining the tendency to acquire electrons from a donor [1]. Redox-active essential metals can therefore act as catalytic cofactors in a wide range of cell enzymes either generating or catalyzing reactive oxygen species (ROS). These species can induce an oxidative stress if they exceed the cell antioxidant capacity damaging cellular proteins, lipids and DNA [21,22]. Beside these effects, an increasing level of ROS further triggers pro-inflammatory signaling cascades into the cell able to induce its programmed death [23]. Therefore, toxicity relates with the aerobic respiration process producing partially reduced forms of molecular oxygen (O_2_), such as hydrogen peroxide (H_2_O_2_) and superoxide (O_2_^•−^). With the presence of some metals, Fenton reactions (see Equation 1) occur intensifying oxygen toxicity by catalyzing the electron transfer from a donor biomolecule to H_2_O_2_ producing hydroxide (OH^−^) and the highly reactive hydroxyl radical (OH^•^):

Fe^2+^ + H_2_O_2_ → Fe^3+^ + OH^−^ + OH^•^ (1)

The presence of external metals can therefore increase the aforementioned reactions producing an excess of ROS derivatives and, as a consequence, an oxidative stress in the cell. Depending on the specific metal involved, at least three mechanisms have been proposed for the ROS increase during metal poisoning in bacteria [1]: (1) acceleration of Fenton reactions [24,25]; (2) disruption of Fe bonded cellular donor ligands, for instance [4Fe–4S] clusters of proteins, resulting in the release of additional Fenton-active Fe [1]; and (3) thiol-mediated reduction leading to the generation of ROS via intermediate S radical chemistry [1].

Regarding Cu-induced cellular toxicity, several mechanisms have been proposed based on the formation of ROS by free Cu ions as both cupric and cuprous ions can participate in redox reactions [26]. In the presence of either superoxide or other reducing agents such as ascorbic acid, Cu^2+^ can be reduced to Cu^+^ catalyzing the formation of hydroxyl radicals from hydrogen peroxide via the Haber-Weiss reactions [27]:

O_2_^•−^ + Cu^2+^ → O_2_ + Cu^+^ (2)

Cu^+^ + H_2_O_2_ → Cu^2+^ + OH^−^ + OH^•^ (3)

The hydroxyl radical is the most powerful oxidizing radical reacting with practically every biological molecule [28]. It can initiate oxidative damage by abstracting the hydrogen both from an amino-bearing carbon to form a carbon centered protein radical and from an unsaturated fatty acid to form a lipid radical.

Although most of the mechanisms explaining the biocidal activity of metals are directly based on the presence of ROS, there are others such as those associated with either the direct redox processes on molecules, for instance the oxidation of cellular thiols, or the indirect formation of ROS [1]. Some metal atoms can form covalent bonds with S leading to the formation of protein disulphides and to the depletion of antioxidant reserves, particularly glutathione, within microbial cells [1]. Metals could further catalyse site-specific damage to cellular proteins by an oxidation process causing loss of catalytic activity and triggering an active process of protein degradation. Finally, there are reports about genotoxicity where, for instance, lethal DNA damage in *Escherichia coli* can be catalyzed by Fe-mediated Fenton chemistry [1].

(b) Mechanisms based on the donor atom selectivity and/or speciation of metals: metal ions in general bind to some atoms of donor ligands, such as O, N and S, through strong and selective interactions [1,29]. Indeed, external metal ions or their complexes can replace original metals present in biomolecules leading to cellular dysfunction [1,30]. This phenomenon is called ionic mimicry or molecular mimicry, depending on whether metal ions or metal complexes are involved. In this way, some metals can promote the destruction of Fe–S clusters, for instance from bacterial dehydratases that is particularly vulnerable to site-specific inactivation by toxic metals [1,29,30,31]. Metals can also replace non-catalytic metal-binding sites inhibiting enzyme activity [1]. Cupric ions (Cu^2+^) in particular are able to form organic complexes with sulfur-, nitrogen- or oxygen-containing functional groups present in the microorganism. This may result in defects in the conformational structure of nucleic acids and proteins, besides changes in oxidative phosphorylation and osmotic balance. Finally, bacteria and yeast exposed to toxic doses of some metals upregulate genes involved in the elimination of ROS generating oxidative stress [1].

Independent on the aforementioned, another possible classification for the mechanisms can be based on where the biocidal metal acts: in the cell membrane or in the intracellular region. The former is based on the fact that bacterial membranes contain macromolecules with highly electronegative chemical groups that serve as sites of adsorption for metal ions [32]. Because of their ability to coordinate metals, it has been postulated that the membrane is the site at which some metals exert bactericidal toxicity [1]. Other evidence suggests that some metals, particularly Ag, disrupt the activity of the bacterial electron transport chain. Ag^+^ can dissipate the chemiosmotic potential of the membrane by causing proton leakage through the membrane [1]. One of the best-known consequences of copper excess is the peroxidative damage to membrane lipids by the reaction of lipid radicals and oxygen [26]. Regarding the intracellular region, metal toxic mechanisms based on either ROS or atoms selectively can occur here where DNA and most proteins are presented. Notably, these two sites for metal toxic action are likely related as recently reported [33]. Under this hypothesis, the cell membrane is first damaged by the metal ions, allowing their subsequent further intake into the intracellular region.

### 2.2. Metal Nanoparticles

During the last years, nanotechnology has produced a new route to take advantage of the antimicrobial behavior of metals by synthesizing highly active metal nanoparticles [29]. Biocidal metal nanoparticles can be either immobilized or coated onto surfaces towards application in several fields such as medical instruments and devices, water treatment, and food processing, among others [30]. However, their combination with polymers forming composites is stressed for a better, and easier, utilization of the antimicrobial activity of these nanoparticles [30].

**Figure 2:**
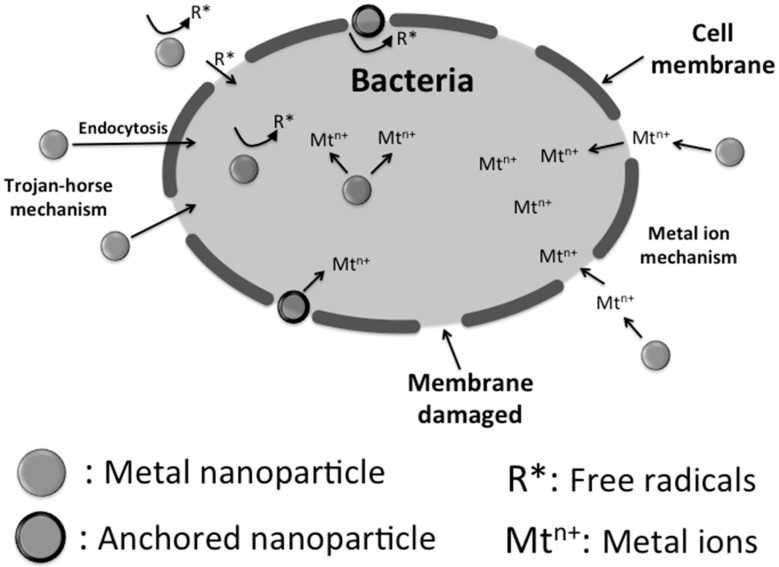
A summary of the mechanisms associated with the antimicrobial behaviour of metal nanoparticles: (1) “Trojan-horse effect” due to endocytosis processes; (2) attachment to the membrane surface; (3) catalyzed radical formation; and (4) release of metal ions.

Nanoparticles can dissolve faster in a given solution volume as compared with larger particles releasing therefore a higher amount of metal ions [34]. Therefore, based on the mechanisms aforementioned based on the presence of metal ion, nanoparticles should present stronger antimicrobial effects than either microparticles or metal surfaces. For instance, the copper nanoparticle corrosion in distilled water is quite different as compared with microparticles [35]. The Cu^2+^ transformation ratio of microparticles increases slowly with the immersion time and levels off eventually, meanwhile in nanoparticles this transformation ratio increases sharply with the immersion time, reaching a peak rapidly, and then decreasing increases sharply with the immersion time, reaching a peak rapidly, and then decreasing. However, new toxic mechanisms, depending on the cellular characteristics at the nano-scale, emerge by taking into account the role of the particle size itself [26]. The best example relate with the direct incorporation of nanoparticles into the cell via endocytotic mechanisms. Afterward the cellular uptake of ions increases as ionic species are subsequently released within the cells by nanoparticle dissolution, a process often referred as “the Trojan horse mechanism” [36,37,38]. This high intracellular concentration gained after nanoparticle dissolution within the cell likely results in massive oxidative stress. A summary of the possible mechanisms associated with the antimicrobial behavior of metal nanoparticles are displayed in Figure 2, and will be discussed below in detail.

#### 2.2.1. Silver Nanoparticles

Silver is generally used in the nitrate form to induce antimicrobial effects, but when silver nanoparticles are used, there is a huge increase in the surface area available opening new approaches [22]. The most pronounced effect of silver nanoparticles is on the cellular metabolic activity and the membrane inflicting damage to the cells and potentially resulting in a myriad of secondary effects, such as generation of ROS and DNA damage [39]. The potency of silver nanoparticles to induce cell damage compared to silver ions is cell type and size-dependent. However, the exact mechanism which silver nanoparticles cause antimicrobial effect is not clearly known although there are several theories about their biocidal action on bacteria. Silver nanoparticles have the ability to anchor to the bacterial cell wall and subsequently penetrate it, causing structural changes in the cell membrane such as in permeability, and afterward cell death [22]. There is also a formation of “pits” on the cell surface increasing the accumulation of silver nanoparticles on the cell surface [40]. The formation of free radicals by silver nanoparticles may also be considered to explain cell death. Electron spin resonance spectroscopy results suggest the formation of free radicals by silver nanoparticles when they are in contact with the bacteria. These radicals can damage the cell membrane and make it porous leading to cell death [22,41,42]. Despite all these mechanisms, the release of silver ions by nanoparticles is also proposed as the main toxic mechanism [43].

#### 2.2.2. Copper Nanoparticles

Micrometric metal copper did not cause cell damage as compared with highly biocidal copper nanoparticles at the same mass [34]. However, when the cells were exposed to the same surface area, the cell membrane damage was similar showing the relevance of this parameter. Cu nanoparticles can be more easily oxidized when interacting with cell membranes containing higher O_2_ concentration as compared with the cell media [34]. In the same article, it was concluded that the cell membrane damage does not relate directly with ionic species but rather with the metal release process at the particle-cell surface interface. However, another work focused on copper oxide (CuO) nanoparticles presented other antecedents [2]. The soluble ions released from the nanoparticles caused cytotoxicity by interacting either directly with the cellular membrane or intracellularly. The biocidal effect of the ions released from CuO nanoparticles was partially explained in amino acid-rich medium through formation of copper-peptide complexes, rather than by the solid nanoparticles. However, the effect of ions from CuO nanoparticles is different as compared with the effect of ions from copper salts, either nitrate or sulfate, at identical soluble concentrations [2]. These results stress the complex behavior of antimicrobial metal nanoparticles as either the particle itself or their ions can participate in the biocide mechanisms. Anyway, independent of that, in the end, metal ions are the active biocidal agent and therefore the same mechanisms detailed in Figure 1 finally explain the antimicrobial effect of metal nanoparticles.

## 3. Polymer/Metal Composites

### 3.1. A General Overview

One of the best methods to further extend the range of applications of antimicrobial metals is by their addition into a polymer obtaining a composite material. However, the best methodology or technology is far to be solved, as it will depend on both the final application and the polymer matrix used. For instance, metals can be either incorporated on the surface of a polymer or embedded into the matrix. In particular, copper has been impregnated on the surface of cotton fibers, latex, and other polymeric materials [44]. These materials present broad-spectrum antimicrobial (*i.e.*, antibacterial, antiviral, and antifungal) and antimite activities. Copper can further be incorporated by plasma immersion ion implantation producing antibacterial polyethylene surface [9]. The production of copper alginate-cotton cellulose (CACC) composite fibers by immersing cotton fibers in aqueous solution of sodium alginate is another approach producing biocidal materials [45]. The process includes the ionic crosslinking of alginate chains within the cotton cellulose fibers with Cu^2+^ ions. Regarding silver, substituted zeolites are one of the most widely-used particles in antimicrobial polymer additives, particularly for food applications [18]. Sodium ions present in zeolites are substituted by silver ions meaning that these particles act as a carrier for antimicrobial ions. The same methodology applies for copper, zinc, and other antimicrobial metals exchanged in zeolites that are later embedded in polymer matrices [46]. Despite these approaches, during the last years the use of metal nanoparticles has gained interest triggered by the amazing properties appearing at the nanoscale, for instance enhanced antimicrobial behavior [40]. In polymer science, the impact of nanotechnology comes from the development and preparation of nanocomposites defined as hybrid materials containing nanometric inorganic fillers embedded into a polymeric matrix.

Polymeric nanocomposites are opening up a new generation of macromolecular materials with low densities and multifunctional properties [47]. The main advantage of nanocomposites is the extremely low amount of filler needed to achieve the desired requirements that can be one or even two orders of magnitude lower than conventional micro-fillers [7,47]. Therefore, in the next sections we will focus on polymer/metal nanocomposites, in particular those using copper and silver as the active agent. We stress however that independent of the metal-based particle used as additive (e.g., metal, nanoparticle, oxide, salt, complex, exchanged zeolite, *etc.*), the mechanism for the antimicrobial behavior of the polymer composite will correspond with the release of metal ions. In this way, the same mechanisms discussed below for polymer/metal nanocomposites can be extended to any polymer/metal antimicrobial material.

**Figure 3:**
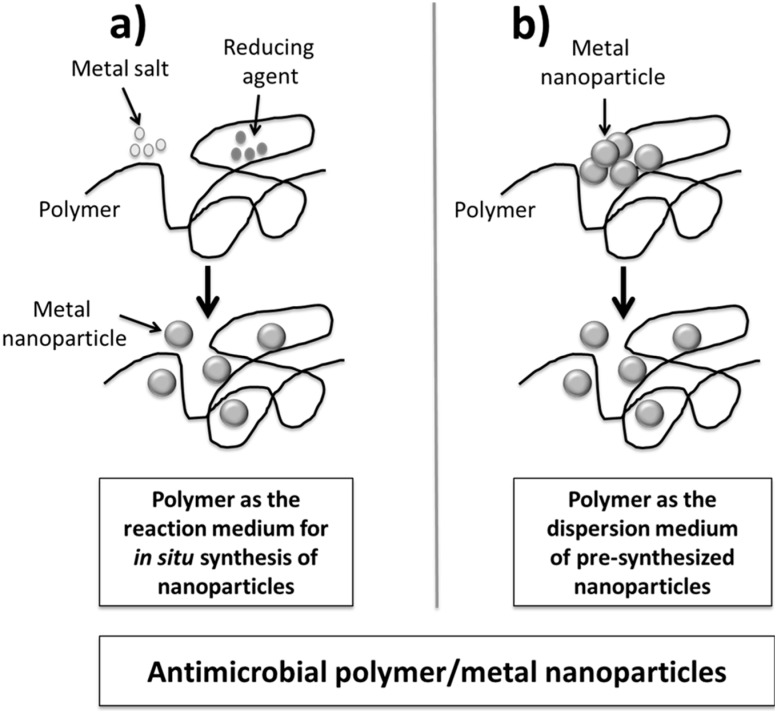
Two main routes producing antimicrobial polymer/metal nanocomposites: (**a**) Polymer as reaction mediun for *in-situ* synthesis of nanoparticles; and (**b**) Polymer as a dispersion mediun of pre-synthesized nanoparticles.

Two general approaches can be distinguished for the preparation of polymer/metal nanocomposites depending on where the nanoparticles are synthesized (Figure 3): (1) *in situ* by using the polymer matrix as the reaction medium; and (2) *ex situ*, meaning that the particle is synthesized before their incorporation into the polymer and in this way the matrix is just the dispersion medium. The former approach is mostly used for polymer hydrogel nanocomposites where the presence in the macromolecules of both several functional groups and a water-rich medium improves the metal stabilization and dispersion. The second approach is currently used in thermoplastic composites where the high viscoelastic matrix at the melt state improves the dispersion of the nanoparticles.

### 3.2. Polymer/Silver Composites

Silver nanoparticles are the most used antimicrobial filler in polymeric nanocomposites [48]. In particular, extensive research has focused on the development of hydrogel composites where silver nanoparticles are *in situ* synthesized as displayed in Figure 3a. In general, all these systems used silver-salt precursors, such as nitrate, together with a reducing agent, in presence of the polymer gel network acting as a nanoreactor where the nanoparticles are formed. For instance, swollen poly (acrylamide-co-acrylic acid) hydrogels can be a medium for the formation of silver nanoparticles with sizes around 25–30 nm [49]. These nanocomposites demonstrated excellent antibacterial activity depending on the nanoparticle size as this parameter changes the surface area that is in contact with the bacterial species. Superabsorbent hydrogel-silver nanocomposite based on poly(vinyl alcohol) and sodium alginate has also been prepared using free radical polymerization [50]. Highly stable silver nanoparticles were *in situ* synthesized in the hydrogel networks by reduction of silver nitrate with sodium borohydride as a reducing agent. The silver nanocomposite hydrogel showed very good antibacterial activity on gram-positive and gram-negative microorganisms. Semi interpenetrating polymer network (IPN) hydrogels, in which poly(vinyl pyrrolidone) chains were physically dispersed throughout poly (acrylamide) gel networks, were synthesized and used as nanoreactors for producing and stabilizing metal nanoparticles [51]. In particular, metal nanoparticles were entrapped throughout hydrogel networks via polyvinylpyrrolidone chains with sizes around 4 nm. These semi-IPN hydrogel-silver nanocomposites presented antibacterial behaviors. The same authors later on developed antimicrobial silver-based composites using semi interpenetrating hydrogel networks based on crosslinked poly(acrylamide) prepared through a redox-solution polymerization in the presence of carbohydrate polymers [52]. In this case, three different polymers were studied: gum acacia, carboxymethylcellulose, and starch. The method allows the synthesis of nanoparticles with sizes between 5 and 20 nm depending on the polymer. All the nanocomposite hydrogels presented similar antimicrobial behavior. Hydrogel networks based on *N*-isopropylacrylamide and sodium acrylate were also prepared by redox-polymerization and used to synthesize highly stable and uniformly distributed silver nanoparticles [53]. Similar to previous works, the hydrogel network is a carrier where the *in situ* reduction of silver nitrate in the presence of sodium borohydride as a reducing agent occurred. These hybrid hydrogels with different sizes of silver nanoparticles can be effectively employed as antibacterial material.

The *in situ* synthesis of silver nanoparticles can also be extended to non-hydrogel based polymers. A simple technique based on the *in situ* synthesis of silver nanoparticles by dissolving silver 1,5-cyclooctadiene-hexafluoroacetylacetonate in amorphous polystyrene has been reported [54]. The metal precursor can thermally decompose producing silver atoms that diffuse into the polymer and form clusters. These silver-doped polystyrenes provide high antibacterial activity. A similar approach was used to add silver nanoparticles into polyamide matrices producing novel antimicrobial materials [7]. In this case, the nanoparticles were first produced in a masterbatch by thermal reduction of silver acetate during melt processing of the matrix. In a second extrusion step, the masterbatch was diluted with pure polyamide. Notably, the antimicrobial behavior of these nanocomposites was compared with polyamide/silver microcomposites. Polyamide filled with just 0.06 wt % silver nanoparticles is able to eliminate the bacteria completely within 24 h whereas microcomposite containing 1.9 wt % of silver kills only about 80% of the bacteria during the same time. The rate of the silver ion release from the nanocomposites is about one order of magnitude higher in comparison to the microcomposites, because of the much larger specific surface area of the nanoparticles [7]. Similar *in situ* routes were further extended by these authors to polyurethane composites and compared with composites based on silver nanoparticles synthesized in invertible polyester prior to their incorporation in the matrix producing smaller particles [55]. At a constant weight percentage of silver in the polymer, the composite with the *ex situ* silver nanoparticles exhibits a silver ion release which is about two orders of magnitude higher than the release from the composite with the *in situ* silver nanoparticles [55]. This observation is explained by the higher specific surface area of the smaller particles and the higher coefficient of diffusion for silver ions. The polymer composite with a concentration of only 0.07 wt % of *ex situ* silver nanoparticles exhibits a high enough release of silver ions to achieve antimicrobial properties.

There are other methods able to produce antimicrobial polymer/silver nanocomposites. For instance, natural sodium alginate polymer acting as both reducing and stabilizing agent can also be used to synthesis silver nanoparticles [56]. The nanoparticles capped with alginate displayed antimicrobial behavior and they were further blended with varying amounts of chitosan forming polyelectrolyte complexes that were casted into stable films. The blended film demonstrated excellent antibacterial activity. Silver nanoparticles can also be synthesized in water by reduction of silver salts in the presence of poly(acrylates) of different molecular weights [57]. These results clearly showed that the reduction method and the polymer chain length played key roles in the achievement of a few-nanometer-sized nanoparticles. The nanoparticle dispersions were then used to functionalize cotton, wool, and polyester samples in order to obtain antimicrobial textiles for biomedical applications. Addition of silver nanoparticles on polymer surfaces by plasma processes is another method reported for the production of antimicrobial materials [58,59]. Deposition of silver nanoparticles onto surface-functional porous poly(ethylene glycol dimethacrylate-co-acrylonitrile) microspheres has also been reported with excellent biocide behaviours [60]. Antimicrobial coatings can further be developed based on plasma polymerized polyacrylic acid (PPAA) deposited on a polyethylene terephthalate mesh [14]. This method allows the entrapment of silver nanoparticle as the carboxylic groups of PPAA can act as anchor as well as capping and stabilizing agents for silver nanoparticles synthesized by a reduction method [14]. Other antimicrobial polymer coatings for a standard metallic orthopaedic substrate containing chitosan, Bioglass^®^ microparticles, and silver nanoparticles, were recently fabricated using a single-step electrophoretic deposition (EPD) technique [13]. The low released concentration of Ag ions (<2.5 ppm) was efficiently antibacterial against *Staphyloccocus aureus* up to 10 days. Polymer coatings with silver nanoparticles can also be electrosynthesised on the surface of titanium based implants by using polyacrylate-based hydrogel starting from poly(ethylene glycol diacrylate)-co-acrylic acid [15]. These hydrogel coatings present swelling capabilities. Silver ion release was properly tuned in order to assure antibacterial activity against the most common pathogens in implant infections while preserving osteoblasts response at the implant interface [15].

### 3.3. Polymer/Copper

Antimicrobial polymer composites based on copper nanoparticles have been much less studied than those based on silver. The reasons might be related to the higher stability and efficiency of silver as compared with copper, or, likely more important, to less available knowledge concerning copper antimicrobial effects. One of the firsts antecedents regarding the development of polymer/copper metal nanocomposites used polyvinylmethyl ketone, poly-(vinyl chloride), and polyvinylidenefluoride as the polymer matrices. These composites were able to show antifungal and bacteriostatic properties depending on the specific properties of the polymeric matrix [61]. The results of the biostatic activity correlate very well with the copper ions released directly into the yeast-free culture broth exposed to the nanocomposites for 4 h. It was also reported that the Cu^2+^ release rate increases with the increasing of mass fraction of copper nanoparticles. Another approach is the attachment on the copper nanoparticle surface of acrylic groups that can be later on copolymerized with other acrylic monomers. These hybrid materials can become an integral part of the polymer backbone and they have a strong potential for use in antibacterial or marine antifouling coatings [62]. Coatings based on polymer/copper nanocomposites provide an interesting route to develop antimicrobial materials [9,12]. Beside plasma-based methodologies previously discussed, poly(ethylene glycol diacrylate) hydrogel thin films can be modified with copper nanoparticles. These coatings were firmly attached on metal substrates by means of electrochemical polymerization technique and present antimicrobial behavior [12].

A relevant set of antimicrobial polymer/copper nanocomposites are based on natural occurring biopolymers such as cellulose. Nanocomposites based on both vegetable and bacterial cellulose matrices were prepared by *in situ* and *ex situ* methods [63]. The results showed that the chemical nature and morphology of the copper nanofillers have great effect on the antibacterial activity, with an increase in the antibacterial activity when the copper content is increased in the composites. The cellulosic matrices also showed an effect on the antibacterial efficiency of the nanocomposites, with vegetal cellulose fibers acting as the most effective substrate [63]. Another study also reports copper nanoparticles in cellulose by modification of its fibers by periodate-induced oxidation followed by covalent attachment of the biopolymer chitosan [64]. The borohydride-induced reduction yielded copper nanoparticle-loaded fibers with an average diameter of particles around 30 nm that showed biocidal action.

Regarding other thermoplastic polymer matrices, the mechanism summarized in Figure 3b was used to produce polyethylene/copper metal nanocomposites by melt blending for intrauterine devices having excellent bioactive properties [65,66]. The introduction of a porous structure can improve the cupric ion release rate of these composites [67]. Polypropylene has also been mixed with copper metal nanoparticles by melt blending in order to produce antimicrobial plastic materials [68,69]. The biocide kinetics can be controlled by the nanofiller content and composites with nanoparticle concentrations higher than 10 *v*/*v* % eliminated 99% of the bacteria in less than 2 h. Copper oxide nanoparticles were also embedded in polypropylene showing stronger antimicrobial behavior than metal copper nanoparticles [69].

A different route is the preparation of hybrid copper nanoparticle fillers such as bentonite supported copper nanoparticles [70]. In an aqueous solution of copper sulfate, the sodium cations within the bentonite interlayers were exchanged for Cu^2+^. These exchanged copper cations were reduced by adding hydrazinium hydrate. Such aqueous bentonite/metal hybrid nanoparticle dispersions were blended with cationic polymethylmethacrylate (PMMA) latex to produce hybrid nanocomposites containing exfoliated polymer grafted organoclay together with bentonite supported metal nanoparticles. The PMMA/bentonite/copper hybrid nanocomposites exhibited high antimicrobial activity.

## 4. Mechanisms for Antimicrobial Polymer/Metal Nanocomposites

The toxic mechanisms of antimicrobial materials coming from the mixture of an antimicrobial agent and a non-active polymer, are as similar as the mechanism of the agent itself [18,48]. In polymer/metal nanocomposites therefore the main toxic mechanism relates with the nanoparticles meaning, as previously discussed, two possible routes depending on the species considered as the active agent: (1) the metal nanoparticle or (2) the metal ions released from the particles [22]. However, a growing number of reports indicate that the ion release is the driving force behind the antimicrobial properties of antibacterial nanoparticles [1,2]. In fact, most of the analyses regarding antimicrobial metal nanoparticles focused on the metal ion release instead of the particle absorbed by the bacteria [30,62,71]. This was confirmed by the results coming from polymer/metal nanocomposites where the antimicrobial effect of these materials related with the metal ion releases rather than with the leaching of the particle [68,69]. The presence of a polymer film covering the nanoparticles as found previously by X-ray photoelectron spectroscopy (XPS) analysis in thermoplastic polymer/copper composites, confirms the hypothesis that the ion release is the main mechanism in this case [68]. However, the exact route for the action of the nanoparticle will depend on the specific characteristics of the polymer used, as in antimicrobial hydrogels the particle release has been stated as the main process for this behavior.

**Figure 4:**
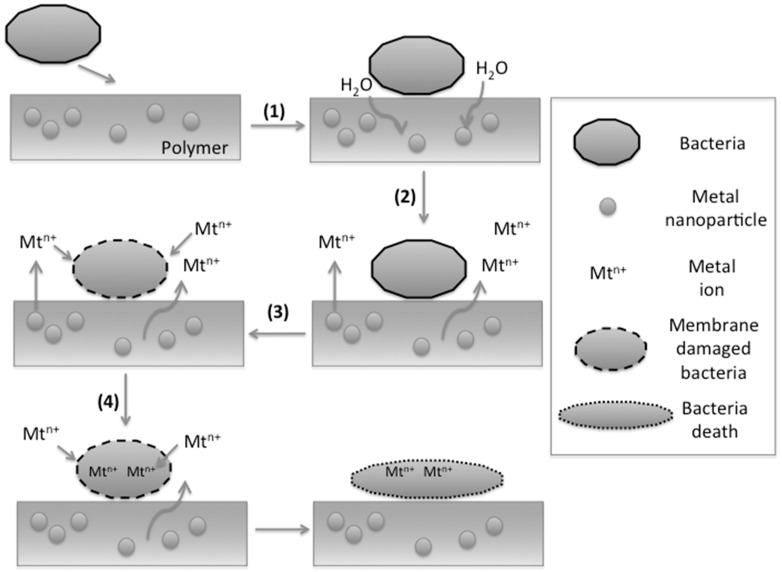
Mechanisms for the antimicrobial behavior of polymer/metal nanocomposites based on thermoplastic matrices: (**1**) adsorbtion of bacteria on the polymer surface triggering the diffusion of water through the polymer matrix due to the medium surrounded the bacteria; (**2**) water with dissolved oxygen reaches the surface of embedded metal nanoparticles allowing dissolution or corrosion processes and in this way metal ions are realized; (**3**) metal ions reach the composite surface damaging the bacteria membrane; (**4**) Afterward, metal ions can diffuse into the bacteria. The details of the specific mechanisms are explained in Figure 1. Although this figure represents a polymer nanocomposite, it can be extrapolated to any polymer/metal materials with the biocide agent embedded in the matrix.

We first focus on the mechanisms for nanocomposites based on thermoplastics, or dense polymers in general, as representing the most complex system from the material point of view as summarized in Figure 4. Afterward, the discussion will be straightforward for nanocompositesbased on hydrogels. As aforementioned, in dense polymers with embedded metal nanoparticles, the ion release is the main mechanism behind their biocide activity. In this context, particles present in the surface-region of the composites seem to be the first approach to understand the experimental data. However, XPSanalysis shows that these nanoparticles are not present on the surface of these samples [68] or at concentrations lower than the bulk of the material [61]. Therefore, the only suitable mechanism for the release of metal ions is the corrosion of particles present in the bulk of the polymer owing to the diffusion of water molecules coming from the bacteria medium into the surface of particles [7]. Even highly non-polar matrices such as polyethylene or polypropylene allow the diffusion of water molecules through [72]. The polymer particle interface can further increase water diffusion through holes or micron-scale defects, allowing fast Knudsen diffusion. This mechanism is directly extended to more polar matrices. When a polyvinylmethyl ketone was used as the polymer matrix, the minimum depth where the nanocomposite becomes hydrated, and eventually releases the soluble copper species, was estimated around 50 nm, which was about 1/10 of the total film thickness in that case [61]. When water with dissolved oxygen reaches the metal particles presented in the polymer bulk, the standard corrosion process occurs [7,68,69]. Afterward, ions coming from corrosion or dissolution process can diffuse-out through the polymer matrix and finally be released. This mechanism is confirmed for metal copper nanoparticles by comparing the X-ray diffraction analysis of the original composite with the diffraction of the same sample but immersed in water. In the latter case, new diffractions peaks appear related with a Cu_2_O layer formed on the surface of the particle [68,69]. All these processes, especially those related with the dissolution step, can be accelerated by the presence of the bacteria in the near-region of the composite due to its organic compounds, pH change, and high surface affinity [2]. In hydrogels the release of nanoparticles from the matrix due to the much larger free volume of these polymers as compared with dense thermoplastic matrices should be further considered [52,53]. Moreover, the diffusion issues of water into/out-to the matrix can be avoided due to the nature of the hydrogel. Therefore, in hydrogels the mechanisms are much closer to pure metal nanoparticles.

## 5. Conclusions

The addition of metal-based particles into polymers is a versatile route to take advantage of their strong antimicrobial properties producing novel biocide materials and allowing a further extension of the range of applications. In this context metal nanoparticles emerge for the production of a broad range of polymer nanocomposites with a high release of metal ions and therefore antimicrobial behaviors. Although there are several examples related with copper and silver nanoparticles embedded into different polymer matrices, as discussed through this manuscript, further research is needed to support the development of novel bioactive polymeric materials to be used in hospital equipment or in prostheses, avoiding for example hospital acquired infections. In this context, we stress technologies based either on commercial matrices with embedded metal nanoparticles or on polymer/metal coatings, as they can be easily implemented at industrial scale or in implant materials, respectively. Future areas of investigations may include scaling up processes, optimization of the filler dispersion and therefore of the ion release, novel metal-based antimicrobial nanoparticles, different polymer matrices such as elastomers, *in situ* studies about the antimicrobial behaviour of the novel materials, and toxicity, among others.

## References

[1] Lemire J.A., Harrison J.J., Turner R.J. (2013). Antimicrobial activity of metals: Mechanisms, molecular targets and applications. Nat. Rev. Microbiol..

[2] Gunawan C., Teoh W.Y., Marquis C.P., Amal R. (2011). Cytotoxic origin of copper(II) oxide nanoparticles: Comparative studies with micron-sized particles, leachate, and metal salts. ACS Nano.

[3] Curtis L.T. (2008). Prevention of hospital-acquired infections: Review of non-pharmacological interventions. J. Hosp. Infect..

[4] Cheadle W.G. (2006). Risk factors for surgical site infection. Surg. Infect..

[5] Sastri V.S. (2010). Plastic in Medical Devices: Properties, Requirements and Applications.

[6] NGP Europe Polyolefins for Use in Medical Devices. http://www.ngphrama.eu.com.

[7] Damm C., Munstedt H., Rosch A. (2008). The antimicrobial efficacy of polyamide 6/silver-nano- and microcomposites. Mater. Chem. Phys..

[8] Kenawy E.R., Worley S.D., Broughton R. (2007). The chemistry and applications of antimicrobial polymers: A state-of-the-art review. Biomacromolecules.

[9] Zhang W., Zhang Y.H., Ji J.H., Zhao J., Yan Q., Chu P.K. (2006). Antimicrobial properties of copper plasma-modified polyethylene. Polymer.

[10] Jones D.S., Djokic J., Gorman S.P. (2005). The resistance of polyvinylpyrrolidone-Iodine-poly(ε-caprolactone) blends to adherence of Escherichia coli. Biomaterials.

[11] Yuan Y.L., Ai F., Zang X.P. (2004). Polyurethane vascular catheter surface grafted with zwitterionic sulfobetaine monomer activated by ozone. Colloid Surf. B.

[12] Cometa S., Iatta R., Ricci M.A., Ferretti C., de Giglio E. (2013). Analytical characterization and antimicrobial properties of novel copper nanoparticles-loaded electrosynthesised hydrogel coatings. J. Bioact. Compat. Polym..

[13] Pishbin F., Mouriño V., Gilchrist J.B., McComb D.W., Kreppel S., Salih V., Ryan M.P., Boccaccini A.R. (2013). Single-step electrochemical deposition of antimicrobial orthopaedic coatings based on a bioactive glass/chitosan/nano-silver composite system. Acta Biomater..

[14] Kumar V., Jolivalt C., Pulpytel J., Jafari R., Arefi-Khonsari F. (2013). Development of silver nanoparticle loaded antibacterial polymer mesh using plasma polymerization process. J. Biomed. Mater. Res. A.

[15] De Giglio E., Cafagna D., Cometa S., Allegretta A., Pedico A., Giannossa L.C., Sabbatini L., Mattioli-Belmonte M., Iatta R. (2013). An innovative, easily fabricated, silver nanoparticle-based titanium implant coating: Development and analytical characterization. Anal. Bioanal. Chem..

[16] Huang J., Murata H., Koepsel R.R., Russell A.J., Matyjaszewski K. (2007). Antibacterial polypropylene via surface-initiated atom transfer radical polymerization. Biomacromolecules.

[17] Lee S.B., Koepsel R.R., Morley S.W., Matyjaszewski K., Sun Y., Russel A.J. (2004). Permanent, nonleaching antibacterial surfaces 1: Synthesis by atom transfer radical polymerization. Biomacromolecules.

[18] Appendini P., Hotchkiss J.H. (2002). Review of antimicrobial food packaging. Innov. Food Sci. Emerg. Technol..

[19] Pehlivan H., Balkose D., Ulku S., Tihminlioglu F. (2005). Characterization of pure and silver exchanged natural zeolite filled polypropylene composite films. Compos. Sci. Technol..

[20] De Azeredo H.M.C. (2009). Nanocomposites for food packaging applications. Food Res. Int..

[21] Shleeva S., Tkac J., Christenson A., Ruzgas T., Yaropolov A.I., Whittaker J.W., Gorton L. (2005). Direct electron transfer between copper-containing proteins and electrodes. Biosens. Bioelectron..

[22] Prabhu S., Poulose E.K. (2012). Silver nanoparticles: Mechanism of antimicrobial action, synthesis, medical applications, and toxicity effects. Int. Nano Lett..

[23] Sintubin L., de Windt W., Dick J., Mast J., van der Ha D., Verstraete W., Boon N. (2009). Lactic acid bacteria as reducing and capping agent for the fast and efficient production of silver nanoparticles. Appl. Microbiol. Biotechnol..

[24] Valko M., Morris H., Cronin M.T.D. (2005). Metals, toxicity and oxidative stress. Curr. Med. Chem..

[25] Stohs S.J., Bagchi D. (1985). Oxidative mechanisms in the toxicity of metal ions. Free Radic. Biol. Med..

[26] Gaetke L.M., Chow C.K. (2003). Copper toxicity, oxidative stress, and antioxidant nutrients. Toxicology.

[27] Bremner I. (1988). Manifestations of copper excess. Am. J. Clin. Nutr..

[28] Buettner G.R., Jurkiewicz B.A. (1996). Catalytic metals, ascorbate and free radicals: Combinations to avoid. Radiat. Res..

[29] Grass G., Rensing C., Solioz M. (2011). Metallic copper as an antimicrobial surface. Appl. Environ. Microbiol..

[30] Ruparelia J.P., Chatterjee A., Duttagupta S.P., Mukherji S. (2008). Strain specificity in antimicrobial activity of silver and copper nanoparticles. Acta Biomater..

[31] Xu F.F., Imlay J.A. (2012). Silver (I), mercury (II), cadmium (II), and zinc (II) target exposed enzymic iron-sulfur clusters when they toxify *Escherichia coli*. Appl. Environ. Microbiol..

[32] Zhang Y.M., Rock C.O. (2008). Membrane lipid homeostasis in bacteria. Nat. Rev. Microbiol..

[33] Mathews S., Hans M., Mücklich F., Solioz M. (2013). Contact killing of bacteria on copper is suppressed if bacterial-metal contact is prevented and is induced on iron by copper ions. Appl. Environ. Microbiol..

[34] Karlsson H.L., Cronholm P., Hedberg Y., Tornberg M., de Battice L., Svedhem S., Wallinder I.O. (2013). Cell membrane damage and protein interaction induced by copper containing nanoparticles—Importance of the metal release process. Toxicology.

[35] Xia X., Xie C., Cai S., Yang Z., Yang X. (2006). Corrosion characteristics of copper microparticles and copper nanoparticles in distilled water. Corros. Sci..

[36] Studer A.M., Limbach L.K., van Duc L., Krumeich F., Athanassiou E.K., Gerber L.C., Moch H., Stark W.J. (2010). Nanoparticle cytotoxicity depends on intracellular solubility: Comparison of stabilized copper metal and degradable copper oxide nanoparticles. Toxicol. Lett..

[37] Wang Z., Li N., Zhao J., White J.C., Qu P., Xing B. (2012). CuO nanoparticle interaction with human epithelial cells: Cellular uptake, location, export, and genotoxicity. Chem. Res. Toxicol..

[38] Cronholm P., Midander K., Karlsson H.L., Elihn K., Odnevall Wallinder I., Möller L. (2011). Effect of sonication and serum proteins on copper release from copper nanoparticles and the toxicity towards lung epithelial cells. Nanotoxicology.

[39] Park M.V.D.Z., Neigh A.M., Vermeulen J.P., de la Fonteyne L.J.J., Verharen H.W., Briedé J.J., van Loveren H., de Jong W.H. (2011). The effect of particle size on the cytotoxicity, inflammation, developmental toxicity and genotoxicity of silver nanoparticles. Biomaterials.

[40] Sondi I., Salopek-Sondi B. (2004). Silver nanoparticles as antimicrobial agent: A case study on *E. coli* as a model for Gram-negative bacteria. J. Colloid Interface Sci..

[41] Kim J.S., Kuk E., Yu K., Kim J.H., Park S.J., Lee H.J., Kim S.H., Park Y.K., Park Y.H., Hwang C.Y. (2007). Antimicrobial effects of silver nanoparticles. Nanomedicine.

[42] Danilcauk M., Lund A., Saldo J., Yamada H., Michalik J. (2009). Conduction electron spin resonance of small silver particles. Spectrochim. Acta Part A.

[43] Feng Q.L., Wu J., Chen G.Q., Cui F.Z., Kim T.N., Kim J.O. (2008). A mechanistic study of the antibacterial effect of silver ions on Escherichia coli and Staphylococcus aureus. J. Biomed. Mater. Res..

[44] Borkow G., Gabbay J. (2004). Copper as a biocidal tool. FASEB J..

[45] Grace M., Chand N., Bajpai S.K. (2009). Copper alginate-cotton cellulose (CACC) fibers with excellent antibacterial properties. J. Eng. Fibers Fabr..

[46] Kaali P., Prez-Madrigal M.M., Strmberg E., Aune R.E., Czél G., Karlsson S. (2011). The influence of Ag^+^, Zn^2+^ and Cu^2+^ exchanged zeolite on antimicrobial and long term *in vitro* stability of medical grade polyether polyurethane. Exp. Polym. Lett..

[47] Paul D.R., Robeson L.M. (2008). Polymer nanotechnology: Nanocomposites. Polymer.

[48] Muñoz-Bonilla A., Fernández-García M. (2012). Polymeric materials with antimicrobial activity. Prog. Polym. Sci..

[49] Thomas V., Yallapu M.M., Sreedhar B., Bajpai S.K. (2007). A versatile strategy to fabricate hydrogel-silver nanocomposites and investigation of their antimicrobial activity. J. Colloid Int. Sci..

[50] Ghasemzadeh H., Ghanaat F. (2014). Antimicrobial alginate/PVA silver nanocomposite hydrogel, synthesis and characterization. J. Polym. Res..

[51] Murthy P.S.K., Mohan Y.M., Varaprasad K., Sreedhar B., Raju K.M. (2008). First successful design of semi-IPN hydrogel-silver nanocomposites: A facile approach for antibacterial application. J. Colloid Int. Sci..

[52] Vimala K., Sivudu K.S., Mohan Y.M., Sreedhar B., Raju K.M. (2009). Controlled silver nanoparticles synthesis in semi-hydrogel networks of poly(acrylamide) and carbohydrates: A rational methodology for antibacterial application. Carbohydr. Polym..

[53] Mohan Y.M., Lee K., Premkumar T., Geckeler K.E. (2007). Hydrogel networks as nanoreactors: A novel approach to silver nanoparticles for antibacterial applications. Polymer.

[54] Palomba M., Carotenuto G., Cristino L., di Grazia M.A., Nicolais F., de Nicola S. (2012). Activity of antimicrobial silver polystyrene nanocomposites. J. Nanomater..

[55] Triebel C., Vasylyev S., Damm C., Stara H., Özpınar C., Hausmann S., Peukert W., Münstedt H. (2011). Polyurethane/silver-nanocomposites with enhanced silver ion release using multifunctional invertible polyesters. J. Mater. Chem..

[56] Sharma S., Sanpui P., Chattopadhyay A., Ghosh S.S. (2012). Fabrication of antibacterial silver nanoparticle—Sodium alginate-chitosan composite films. RSC Adv..

[57] Falletta E., Bonini M., Fratini E., Lo Nostro A., Pesavento G., Becheri A., Lo Nostro P., Canton P., Baglioni P. (2008). Clusters of poly(acrylates) and silver nanoparticles: Structure and applications for antimicrobial fabrics. J. Phys. Chem. C.

[58] Jiang H., Manolache S., Wong A.C.L., Denes F.S. (2004). Plasma-enhanced deposition of silver nanoparticles onto polymer and metal surfaces for the generation of antimicrobial characteristics. J. Appl. Polym. Sci..

[59] Del Nobile M.A., Cannarsi M., Altieri C., Sinigalia M., Favia P., Iacoviello G., D’Agostino R. (2004). Effect of Ag-containing nano-composite active packaging system on survival *of Alicyclobacillus acidoterrestris*. J. Food Sci..

[60] Kim J.W., Lee J.E., Kim S.J., Lee J.S., Ryu J.H., Kim J., Han S.H., Chang I.S., Suh K.D. (2004). Synthesis of silver/polymer colloidal composites from surface-functional porous polymer microspheres. Polymer.

[61] Cioffi N., Torsi L., Ditarantano N., Tantalillo G., Ghibelli L., Sabbatini L., Bleve-Zacheo T., D’Alessio M., Zambonin P.G., Traversa E. (2005). Copper nanoparticle/polymer composites with antifungal and bacteriostatic properties. Chem. Mater..

[62] Anyaogu K.C., Fedorov A.V., Neckers D.C. (2008). Synthesis, characterization, and antifouling potential of functionalized copper nanoparticles. Langmuir.

[63] Pinto R.J., Daina S., Sadocco P., Pascoal Neto C., Trindade T. (2013). Antibacterial activity of nanocomposites of copper and cellulose. Antibacterial Activity of Nanocomposites of Copper and Cellulose. Biomed. Res. Int..

[64] Mary G., Bajpai S.K., Chand N. (2009). Copper(II) ions and copper nanoparticles-loaded chemically modified cotton cellulose fibers with fair antibacterial properties. J. Appl. Polym. Sci..

[65] Cai S., Xia X., Xie C. (2005). Corrosion behaviour of copper/LDPE nanocomposites in simulated uterine solution. Biomaterials.

[66] Xu T., Lei H., Cai S.Z., Xia X.P., Xie C.S. (2004). The release of cupric ion in simulated uterine: New material nano-Cu/low-density polyethylene used for intrauterine devices. Contraception.

[67] Zhang W., Xia X., Qi C., Xie C., Cai S. (2012). A porous Cu/LDPE composite for copper-containing intrauterine contraceptive devices. Acta Biomater..

[68] Palza H., Gutiérrez S., Delgado K., Salazar O., Fuenzalida V., Avila J., Figueroa G., Quijada R. (2010). Toward tailor-made biocide materials based on polypropylene/copper nanoparticles. Macromol. Rapid Commun..

[69] Delgado K., Quijada R., Palma R., Palza H. (2011). Polypropylene with embedded copper metal or copper oxide nanoparticles as a novel plastic antimicrobial agent. Lett. Appl. Microbiol..

[70] Weickmann H., Tiller J.C., Thomann R., Mulhaupt R. (2005). Metallized organoclays as new intermediates for aqueous nanohybrid dispersions, nanohybrid catalysts and antimicrobial polymer hybrid nanocomposites. Macromol. Mater. Eng..

[71] Ren G., Hub D., Cheng E.W.C., Vargas-Reus M.A., Reipd P., Allaker R.P. (2009). Characterisation of copper oxide nanoparticles for antimicrobial applications. Int. J. Antimicrob. Agents.

[72] Ton-That T.M., Jungnickel B.J. (1999). Water diffusion into transcrystalline layers on polypropylene. J. Appl. Polym. Sci..

